# Simultaneous Phacoemulsification and Graft Refractive Surgery in Penetrating Keratoplasty Eyes

**DOI:** 10.5402/2011/495047

**Published:** 2011-09-20

**Authors:** Sepehr Feizi, Mohammad Zare, Bahram Einollahi

**Affiliations:** Ophthalmic Research Center, Department of Ophthalmology, Labbafinejad Medical Center, Shahid Beheshti University of Medical Sciences, Tehran 16666, Iran

## Abstract

*Purpose*. To report outcomes of graft refractive surgery (GRS) along with clear-cornea phacoemulsification and intraocular lens (IOL) implantation in penetrating keratoplasty (PKP) eyes. *Methods*. Fourteen eyes of 13 patients who had received PKP underwent simultaneous GRS (relaxing incisions with or without counter-quadrant compression sutures) and clear-cornea phacoemulsification with IOL implantation. To calculate IOL power, preoperative keratometry readings and the SRK-T formula were used. *Results*. Mean patient age and follow-up period were 50.5 ± 14.4 years and 14.6 ± 7.1 months, respectively. A significant increase was observed in best spectacle-corrected visual acuity (from 0.55 ± 0.18 logMAR to 0.33 ± 0.18 logMAR, *P* = 0.001). There was a significant decrease in vector keratometric astigmatism by 6.22 D (*P* = 0.03). Spherical equivalent refraction was reduced from −3.31 ± 3.96 D to −1.69 ± 2.38 D (*P* = 0.02) which did not significantly differ from the target refraction (−0.76 ± 0.14 D, *P* = 0.20). No complications developed and all the grafts remained clear at the final examination. *Conclusion*. Simultaneous phacoemulsification and GRS is a safe and effective method to address post-PKP astigmatism and lens opacity. IOL power can be calculated from preoperative keratometry readings with an acceptable accuracy. However, patients should be informed about the possibility of high refractive errors postoperatively.

## 1. Introduction

The most common complication of penetrating keratoplasty (PKP) is astigmatism [1]. Several studies indicate that 15–31% of the patients who undergo PKP may develop postoperative astigmatism greater than 5 diopters (D) [2–4]. Graft refractive surgery (GRS) consisting of relaxing incisions at steep meridians with or without counter-quadrant compression sutures can significantly reduce post-PKP astigmatism and alter graft steepness [5]. In the majority of cases, GRS is the only procedure performed at a single time. However, it is sometimes combined with other interventions such as cataract extraction and posterior chamber intraocular lens (IOL) implantation to simultaneously address lens opacity and high corneal graft astigmatism. The former develops after corneal transplantation at a higher rate as compared to normal populations due to intraoperative lens trauma, postoperative inflammation, and topical steroid use [6]. 

A stepwise intervention in which GRS is performed first, followed by cataract extraction and IOL implantation after a few months when refraction becomes stable, can also be considered in patients developing both complications. This sequential surgery is more expensive and delays visual rehabilitation. Other options are toric IOL implantation at the time of cataract surgery or laser refractive surgery thereafter. 

The advantages of GRS coupled with cataract extraction are correcting the astigmatism in a single-stage surgery without additional cost and addressing the astigmatism at its origin while preserving the original graft profile. Since graft refractive power and possibly optical chamber depth and axial length are changed by GRS, however, accurate IOL power calculation can be a matter of concern in such a combined intervention [5]. The aim of the present study is to report clinical outcomes of the combined surgery of phacoemulsification and GRS and determine the accuracy of IOL power calculation using preoperative keratometry readings in a group of patients who had developed significant cataract and post-PKP astigmatism.

## 2. Patients and Methods

In this retrospective interventional case series, 14 eyes of 13 patients (6 men and 7 women), aged between 14 and 60 years (mean, 50.5 ± 14.4 years), that underwent simultaneous cataract and GRS between October 2003 and July 2010 were included. The participants had all sutures removed at least 3 months before entering the study. The primary indications for corneal transplantation were keratoconus (10 eyes), macular corneal dystrophy (1 eye), lattice corneal dystrophy (1 eye), chemical burn (1 eye), and old corneal scar (1 eye). Preoperatively, a comprehensive ocular examination including uncorrected and best spectacle-corrected visual acuity (UCVA and BSCVA, resp.), manifest refraction, keratometry using a manual Javal-Schiötz keratometer (Topcon, Capelle a/d IJssel, Netherlands), slit-lamp examination, intraocular pressure measurement, and dilated funduscopy was performed. To determine the exact location of steep meridians, computerized corneal topography analysis (TMS-1 Topographic Modeling System, version 1.61; Computed Anatomy Inc., NY, USA) was used, and the IOL power was calculated by an A-scan ultrasound device (Storz Omega Compu-Scan Biometric Ruler, Storz International, St Louis, MO, USA) using the SRK-T formula in all cases. For IOL power calculation, preoperative keratometry readings measured by the manual keratometer were considered, and postoperative refraction was targeted at −0.76 ± 0.14 D, on average (range, −0.94 to −0.50 D). 

### 2.1. Surgical Technique

All patients underwent surgery under retrobulbar anesthesia. The indication and technique of GRS was previously described [5]. Briefly, the 6 o'clock semimeridian was marked with the patient sitting upright. Then, relaxing incisions were created in the recipient-donor interface down to Descemet's membrane on one or both sides of the steepest meridian before cataract surgery, when the globes were still formed. The arc length of incisions was decided based on the extension of red area on topography. When the topography demonstrated steepening of a hemimeridian, one incision was made on the steep side. But, when the topographic pattern was symmetric or asymmetric bowtie, both sides of the meridian were incised. 

This step was followed by the creation of a single-plane 2.8-mm clear corneal incision just in front of the vascular arcade. As a 2.8-mm clear corneal tunnel at considerable distance from the recipient-donor interface should have no impact on graft astigmatism, the site of main incisions was selected irrespective of the steep meridians, superotemporal in right eyes and superonasal in left eyes. The clear corneal tunnel, intended to be 1.5 to 2.0 mm in length, entered the anterior chamber before reaching the recipient-donor interface. After the injection of a dispersive ophthalmic viscosurgical device (Coatel, Bausch & Lomb, Waterford, Ireland), a 5.0- to 5.5-mm central continuous capsulorhexis was created, and phacoemulsification was performed using the divide and conquer technique. This step was followed by cortical cleanup and implantation of a foldable one-piece monofocal IOL (AcrySof SN60WF, Alcon Laboratories Inc., Fort Worth, Tex, USA) in the capsular bag using the C cartridge (Alcon Laboratories Inc.). At the conclusion of phacoemulsification, the anterior chamber was formed and the clear corneal incision hydrated to become self-sealed. Then, intraoperative keratoscopy was performed using a handheld keratoscope, and interrupted 10-0 nylon counter-quadrant compression sutures were added in the recipient-donor interface if round keratoscopic mire was not achieved by relaxing incisions alone, and the axis of preoperative astigmatism remained unchanged (12 eyes). In these cases, the tension of compression sutures was adjusted to have an astigmatism of 4-5 D at the sutured meridian. These sutures were selectively removed, based on the keratometry readings, starting 2 weeks after surgery, and were completed over 4 to 6 weeks.

### 2.2. Postoperative Course

Postoperatively, the patients were medicated with sulfacetamide 10% eye drops every 6 hours and betamethasone 0.1% eye drops every 3 hours. The antibiotic drop was discontinued after 10 days, while the corticosteroid drop was tapered off over 4 to 6 weeks. Follow-up examinations were performed at days 1, 3, and 7, and months 1, 3, and 6, and then repeated every 6 months. During followup examinations, UCVA, BSCVA, keratometry, manifest refraction, and intraocular pressure were reevaluated.

### 2.3. Statistical Analysis

Data were analyzed using SPSS (v15) statistical software (SPSS Inc., Chicago, Il, USA). Normality was checked by the Kolmogorov-Smirnov test, and normally distributed data were expressed as mean ± standard deviation. Refractive and keratometric spherical equivalent (defined as sphere + (1/2) cylinder and flat keratometry reading + (1/2) keratometric astigmatism, resp.) and astigmatisms were compared before and after GRS using the paired *t*-test. A *P*-value less than 5% was considered statistically significant.

Changes in astigmatism were evaluated by vector analysis [7] and simple subtraction methods in plus cylinder format. Two quantities, including coupling ratio (CR; the ratio of flattening of the incised meridian to steepening of the opposite meridian) and coupling constant (CC; the ratio of change in spherical equivalent to the magnitude of vector change in astigmatism) were calculated, on the basis of keratometry as follows [8]:

(1) $$\begin{array}{c} \text{CR}=\frac{\left(A/2+\Delta \text{SE}\right)}{\left(A/2-\Delta \text{SE}\right)},\\ \text{CC}=\frac{1}{2}\times \frac{\left(\text{CR}-1\right)}{\left(\text{CR}+1\right)}, \end{array}$$

where *A* is the magnitude of vector change in astigmatism in plus cylinder format calculated by the vector analysis method [7] and ΔSE is change in spherical equivalent.

## 3. Results

The mean followup period after PKP was 145.2 ± 54.1 months (range, 26 to 373 months) and after combined phacoemulsification, and GRS was 14.6 ± 7.1 months (range, 4 to 64 months). Mean preoperative UCVA and BSCVA was 1.15 ± 0.24 logMAR (range, 0.70 to 1.60 logMAR) and 0.55 ± 0.18 logMAR (range, 0.30 to 0.80 logMAR), respectively. Mean recipient and donor trephine size were 7.75 ± 0.42 mm (range, 7.5 to 8.25 mm) and 8.10 ± 0.34 mm (range, 7.75 to 8.5 mm), respectively, with a recipient-donor disparity of 0.25 mm in 10 eyes and 0.50 mm in 4 eyes. The IOL power varied from +8.0 to +25.0 D, averaging +17.64 ± 5.39 D. Postoperatively, mean UCVA and BSCVA significantly increased to 0.50 ± 0.19 logMAR (range, 0.30 to 1.0 logMAR; *P* < 0.001) and 0.33 ± 0.18 logMAR (range, 0.18 to 0.80 logMAR; *P* = 0.001), respectively. Of the 14 eyes, 9 (64.3%) had a postoperative UCVA ≥* *20/50 and 9 (64.3%) achieved a postoperative BSCVA ≥* *20/40, with an improvement of two lines or more in Snellen BSCVA in 50% of the cases. Mean postoperative intraocular pressure was 14.6 ± 4.7 mmHg (range, 11.0 to 21.0 mmHg) which did not significantly differ from the preoperative value (13.8 ± 2.5 mmHg; range, 10.0 to 19.0 mmHg; *P* = 0.51).

Table 1 compares pre- and postoperative refractive and keratometric spherical equivalent and astigmatism. Mean reduction in refractive astigmatism was 2.03 D by the subtraction and 5.19 D by the vector analysis method. Corresponding figures for keratometric astigmatism were 2.39 D and 6.22 D, respectively. 

**Figure 1:**
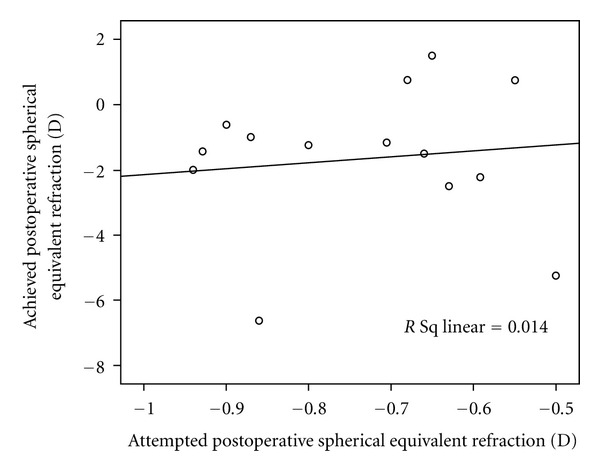
Scattergram demonstrates achieved versus attempted postoperative refraction.

The mean achieved postoperative spherical equivalent refraction did not significantly differ from the mean target refraction (−1.69 ± 2.38 D versus −0.76 ± 0.14 D, resp.; *P* = 0.20).  Figure 1 demonstrates achieved versus attempted postoperative spherical equivalent refraction in individual participants. The absolute value of the difference between these values ranged from 0.13 D to 5.77 D (mean, 1.80 ± 1.74 D). In 4 (28.6%) eyes, postoperative refraction was within ±0.5 D of the target refraction, whereas 5 (35.7%) and 11 (78.6%) eyes had a postoperative refraction within ±1.0 D and ±2.0 D of the expected value, respectively. 

Manifest refraction showed a significant reduction in myopia, postoperatively. However, there was a myopic shift in mean spherical equivalent on the basis of keratometry (0.62 ± 1.80 D) which did not reach a significant level (*P* = 0.26). The insignificant change in overall graft curvature was consistent with the CR close to 1 and the CC near 0 (Table 2). 

No significant complications including inadvertent penetration into the anterior chamber at the sites of the relaxing incisions, vitreous loss, postoperative uveitis, or endophthalmitis developed. The haze produced by the relaxing incisions intraoperatively was not considerable enough to hinder the procedure of phacoemulsification. No episodes of subepithelial or endothelial graft rejection occurred during the followup period. All grafts remained clear throughout the followup period, and no patient experienced any other severe complications.

## 4. Discussion

The most common complication after PKP is astigmatism [1]. Previous studies have demonstrated that GRS consisting of relaxing incisions, with or without compression sutures, is a safe and effective option for reducing high post-keratoplasty astigmatism which develops in 15% to 31% of the patients [2–4]. Using subtraction or vector analysis methods to calculate the reduction in astigmatism, a wide range of correction between 3.4 D and 9.7 D has been reported by several studies employing GRS for post-PKP astigmatism [5, 9–13]. A decrease of 6.22 D in vector keratometric astigmatism observed in this study supports the results of the aforementioned studies.

PKP can accelerate cataract formation, particularly in patients over 50 years of age. Previous studies have shown that 44 to 64% of the patients develop cataracts within 5 years of penetrating keratoplasty, which is significantly higher than the rate in the general population at the same age [6, 14–17]. This higher incidence of cataract formation after PKP can be attributed to intraoperative lens trauma, postoperative inflammation, and topical steroid use. Cataract developing after keratoplasty can be managed safely using advanced phacoemulsification techniques and IOL implantation, with no increased risk of endothelial cell loss, endothelial graft rejection, or failure [15–17]. 

To our knowledge, this is the first study to analyze the safety and efficacy, as well as the clinical outcomes of combined phacoemulsification and GRS after PKP and evaluate reliability of the IOL power calculated on the basis of preoperative keratometry readings. The results of the current study indicate that GRS can be safely combined with phacoemulsification and IOL implantation to simultaneously address both significant lens opacity and graft astigmatism. Other options for these patients are a sequential approach including GRS followed by phacoemulsification, photoastigmatic keratectomy after cataract surgery, and toric IOL implantation [18]. Compared to these methods, GRS has the advantage of low cost and feasibility for both patients and surgeons and can be performed at the time of cataract surgery without requiring any specific instruments. Additionally, in comparison to the nonsimultaneous procedure in which GRS is carried out first, followed by cataract extraction and IOL implantation when the graft curvature becomes stable, the simultaneous approach is completed in a single session, so it provides rapid visual rehabilitation. 

In the present study, there was a significant increase in UCVA (6 lines of the Snellen visual acuity chart, on average) and BSCVA (3 lines) as well as a significant decrease in both manifest refraction and keratometric astigmatism. Additionally, no complications developed intraoperatively or postoperatively, and all grafts remained clear at the final followup examination, which means phacoemulsification and intraocular lens implantation neither affects endothelial cell count, nor increases the rate of future graft rejection episodes [16]. 

In spite of these advantages, however, there is an important drawback for the simultaneous approach, which is the inability to precisely determine the IOL power. Accurate IOL power prediction in standard cataract surgery requires valid and reproducible biometric data pertaining to the corneal curvature, anterior chamber depth, and axial length. In contrast to standard cataract surgery, GRS combined with phacoemulsification and IOL implantation may significantly alter these factors, as graft curvature and perhaps anterior chamber depth and axial length change in an unpredictable manner which could make it difficult to calculate the IOL power accurately. The results of the current study demonstrate that graft astigmatism significantly decreased, whereas keratometry spherical equivalent and mean keratometry increased. However, the increase in graft curvature was not statistically significant, which is attributable to the coupling ratio close to 1 (0.70 ± 1.24) and coupling constant close to 0 (0.04 ± 0.58), indicating that the flattening of the steep meridians was roughly equal to the steepening of the flat meridian. This explains why 78.6% of the eyes had a postoperative refraction within ±2.0 D of the expected value, and the postoperative spherical equivalent refractive error did not significantly differ from targeted values (−1.69 ± 2.38 D versus −0.76 ± 0.14 D, resp.), despite use of preoperative keratometry values. 

However, it should be noted that the absolute difference between the postoperative spherical equivalent and target refraction varied substantially from 0.13 D to 5.77 D, and a residual refractive error of >−5 D was observed in 2 eyes. These changes in graft refractive power, as well as possible alterations in anterior chamber depth and/or axial length can be the reason for unexpectedly high refractive outcomes in some cases.

The results of the present study should be interpreted in the context of its limitations. First, the sample size is small. The second limitation arises from the design of the study which was retrospective. A prospective comparative study recruiting a control arm to receive other interventions (toric IOL implantation, sequential GRS and phacoemulsification, and laser keratorefractive surgery after cataract extraction) can help find the best approach. Third, the time of followup was short, and regression of astigmatism may occur after long-term followup. Forth, we relied only on changes in anterior graft curvature. To better understand the effect of GRS on the IOL power prediction, a study is now underway in our center to determine any alterations in anterior chamber depth, total axial length as well as posterior graft curvature after GRS.

## 5. Conclusion

In conclusion, the present study found combined phacoemulsification with IOL implantation and GRS a safe and effective method in PKP eyes with significant lens opacity and graft astigmatism. In spite of significant change in the graft astigmatism and some increase in the graft steepness, the IOL power can be calculated with acceptable certainty using preoperative keratometry readings and the SRK-T formula. However, patients should be informed about the possible postoperative high refractive error necessitating additional surgical intervention. A prospective study on a larger sample is necessary to achieve a better conclusion.

## Figures and Tables

**Table 1:** Refractive and keratometric spherical equivalent and astigmatism before and after combined phacoemulsification and graft refractive surgery.

Characteristics		Preoperative values mean ± SD (range)	Postoperative values mean ± SD (range)	*P*-value
Spherical equivalent (D)	Refraction	−3.31 ± 3.96 (−9.0 to 2.75)	−1.69 ± 2.38 (−6.75 to 1.50)	0.02
	Keratometry	44.79 ± 2.08 (41.75 to 53.25)	45.65 ± 1.86 (41.0 to 56.50)	0.26

Astigmatism (D)	Refraction	5.43 ± 1.50 (1.75 to 7.0)	3.42 ± 1.73 (1.25 to 6.0)	0.04
	Keratometry	8.10 ± 2.50 (3.50 to 11.50)	5.07 ± 2.53 (2.0 to 9.50)	0.03

Mean keratometry (D)		47.17 ± 3.11 (41.75 to 53.25)	47.79 ± 4.21 (41.0 to 56.50)	0.26

D: diopter; SD: standard deviation.

**Table 2:** Coupling ratio (CR) and coupling constant (CC) calculated on the basis of keratometry.

Characteristic	Mean ± SD (range)
Flattening of incised meridian (D)	2.49 ± 2.86 (−2.50 to 6.95)
Steepening of opposite meridian (D)	3.73 ± 2.78 (−1.50 to 8.02)
CR	0.70 ± 1.24 (−1.33 to 3.56)
CC	0.04 ± 0.58 (−0.67 to 1.75)

D: diopter; SD: standard deviation.
